# Amplicon-based DNA sequencing to characterize Duffy antigen polymorphisms and analysis of Duffy blood system and glucose-6-phosphate dehydrogenase deficiency in Mauritania

**DOI:** 10.1371/journal.pntd.0013882

**Published:** 2025-12-26

**Authors:** Albin Fontaine, Oum Kelthoum Mamadou Djigo, Nicolas Gomez, Ali Ould Mohamed Salem Boukhary, Leonardo Basco, Sébastien Briolant

**Affiliations:** 1 Unité Parasitologie et Entomologie, Département des risques vectoriels, Institut de Recherche Biomédicale des Armées (IRBA), Marseille, France; 2 Aix Marseille Université, Service de Santé des Armées (SSA), Assistance Publique – Hôpitaux de Marseille (AP-HM), Unité Mixte de Recherche (UMR) D 257 Risques Infectieux, Microorganismes Emergents (RITMES), Marseille, France; 3 Institut Hospitalo-Universitaire (IHU) – Méditerranée Infection, Marseille, France; 4 Unité de Recherche Génomes et Milieux, Faculté des Sciences et Techniques, Université de Nouakchott, Nouakchott, Mauritania; 5 Institut de Recherche pour le Développement (IRD), Marseille, France; Advanced Centre for Chronic and Rare Diseases, INDIA

## Abstract

**Background:**

Both Duffy blood antigen expression and G6PD deficiency are known to be associated with ethnic origin. Updates in epidemiological data on the prevalence of polymorphisms in these two human genes are key information for guiding national programs to eliminate *Plasmodium vivax* malaria.

**Methods:**

Duffy genotypes and their predicted phenotypes were determined in 943 blood samples from Mauritanian patients belonging to different ethnic groups (n = 432 White Moors and n = 511 individuals of black African ancestry) with a known G6PD genotype determined by PCR-restriction fragment length polymorphism in our previous study, using a cost-saving multiplexed barcoding technique that allows simultaneous analysis of a large number of samples and next-generation DNA sequencing (NGS).

**Results:**

Duffy-negative phenotype predicted from Duffy genotype was predominant in individuals with black African ancestry (65–88%), while 16% of white Moors were Duffy-negative. Among 432 samples with interpretable Duffy sequence data from white Moors, 7/356 (2.0%) were Duffy-positive and G6PD A^–^ deficient; 8/76 (10.5%) were Duffy-negative and G6PD A^–^ deficient, mostly (n = 6) in heterozygous females. By contrast, among 511 patients of black African ancestry, 13 (13/140, 9.3% including heterozygous females) were Duffy-positive and G6PD A^–^ deficient; 65 (65/371, 17.5%) were Duffy-negative and G6PD A^–^ deficient, mostly (n = 44) in heterozygous females.

**Conclusion:**

A large majority of white Moors are Duffy-positive and susceptible to *P. vivax* infection, but most are eligible for anti-hypnozoite therapy with primaquine at the standard dose. About 15.4% of individuals with black African ancestry were affected by G6PD A^–^ deficiency, independently of their Duffy receptor status. This population requires G6PD screening before primaquine therapy in rare cases of *P. vivax* infection. These results provide important clues about the feasibility to implement an efficient anti-hypnozoite treatment in Mauritania and identify priority areas for targeted interventions against *P. vivax* malaria.

## Author summary

Despite progress achieved in controlling malaria, several challenges remain, including *Plasmodium vivax* malaria. In most cases, this malaria species infects humans with the blood receptor called Duffy antigen (referred to as “Duffy-positive” individuals). After the initial infection, some *P. vivax* parasites (called hypnozoites) have the peculiarity of remaining dormant in the liver cells of incompletely treated patients for months or years before ‘waking up’ to cause other blood infections (called relapse) and spreading the disease. There is an effective medication to kill hypnozoites, but this pill can cause serious side effects in persons with insufficient activity of the enzyme called glucose-6-phosphate dehydrogenase (G6PD) due to mutations. Mutations that influence G6PD activity and determine the presence (or absence) of Duffy blood antigen depend largely on ethnic groups (and sex, for G6PD). Such mutations, in turn, affect the medical decision on whether anti-hypnozoite medication can be given or not to prevent relapse. We studied the proportions of Duffy-positive and Duffy-negative individuals to predict which ethnic groups are more susceptible to develop *P. vivax* and further predict the proportion of patients who can receive anti-hypnozoite medication safely in Mauritania. This epidemiological study attempted to identify which individuals should receive anti-hypnozoite treatment with priority.

## Introduction

Despite overall global progress made towards disease control and even elimination in a few countries over the past two decades, malaria remains a leading cause of morbidity and mortality, particularly among young children and pregnant women in Africa [1]. Effective disease control interventions include the use of rapid diagnostic tests for malaria, artemisinin-based combination therapies (ACT) for treatment, preventive antimalarial drug administration in African children and pregnant women, antimalarial vaccine in African children, distribution of bed nets to prevent mosquito bites, and various chemical means to kill mosquitoes. These disease control measures are directed against *Plasmodium falciparum* [2]. On the global scale, these interventions have also been beneficial in reducing the malaria burden due to *Plasmodium vivax*, another human malaria species with a wider geographical distribution than *P. falciparum*, leading to a considerable decrease in the number of *P. vivax* cases over the past two decades [1]. *Plasmodium vivax* malaria occurs in Asia, in South and Central America, in the Near East, in the Oceania region, as well as in some African countries [3–10].

As many countries adopt measures to control and eliminate malaria, concerns have been raised about *P. vivax* malaria, often considered a neglected disease [3–7]. These concerns are warranted, based on established knowledge and recent findings. First, contrary to long-held beliefs, *P. vivax* is widespread in Africa, including areas where it was thought to be non-existent [8–10]. The misconception that *P. vivax* is absent from sub-Saharan Africa stems from the “Duffy-malaria hypothesis:” most individuals of black African origin are resistant to *P. vivax* infection due to the absence of the Duffy antigen, also known as the “Duffy antigen receptor for chemokines” (DARC), on the surface of erythrocytes [11,12]. In contrast, populations of non-black African origin generally have a Duffy-positive phenotype, making them susceptible to *P. vivax* infection. Duffy antigen has been considered the major receptor for the interaction between Duffy binding protein (DBP) of *P. vivax* merozoites and host reticulocytes during erythrocyte invasion [13], as supported by the general observation that Duffy-negative phenotype offers relative protection from *P. vivax* infection. Second, in Asian and South American countries where *P. falciparum* and *P. vivax* co-exist, the prevalence of *P. falciparum* has been decreasing faster than that of *P. vivax*, leading to an increasing proportion of *P. vivax* cases [14,15]. Third, the now outdated notion of *P. vivax* infection as a “mild” disease has been overcome. It is now recognized that *P. vivax* malaria can cause severe and complicated illness, potentially leading to a fatal outcome [16–19]. Fourth, *P. vivax* (and also *P. ovale*) is distinguished by unique biological features, the most significant for malaria control being its ability to produce hypnozoites during the hepatic stage [20,21]. These dormant hepatic forms can reactivate weeks, months, or even years after the primo-infection, causing new blood infections called relapse [20,21]. Fifth, the only effective drugs that can kill hypnozoites, 8-aminoquinolines (e.g., primaquine and tafenoquine), are still largely underused and/or misused in treating *P. vivax* and *P. ovale* infections. This is either due to unavailability of these drugs in many African pharmacies or poor patient compliance with the standard 14-day treatment regimen [22–26]. As a consequence, many patients with laboratory-confirmed *P. vivax* malaria are not receiving the full treatment recommended by the World Health Organization (WHO), which includes both blood schizonticides (chloroquine or ACT) and anti-hypnozoite drug, to achieve “radical cure,” i.e., the elimination of both blood-stage and latent liver-stage parasites [2,27]. After incomplete treatment, these patients become potential reservoirs and sources of new infections, thereby hampering efforts to interrupt disease transmission.

The major problem with the administration of 8-aminoquinolines as anti-hypnozoite drugs is their potentially serious adverse effects, in particular acute hemolytic anemia, that may occur in patients with glucose-6-phosphate dehydrogenase (G6PD) deficiency [28]. *Plasmodium vivax*-infected patients should be screened and assessed for the level of G6PD enzymatic activity before prescribing primaquine or adjusting primaquine dose in individuals with mild to moderate G6PD deficiency. To promote, facilitate, and enhance the feasibility of primaquine administration in point-of-care settings, several on-the-spot field diagnostic tests for G6PD activity have been developed and assessed in the field [29–33].

G6PD deficiency is an X-linked genetic disorder and is therefore more commonly expressed in men who are hemizygous [34]. In addition to sex, ethnic origin is a major factor that determines not only G6PD deficiency, but also Duffy-negative genotype and phenotype. Malaria has been the selective force for G6PD deficiency (for both *P. falciparum* and *P. vivax*) and Duffy-negative phenotype (for *P. vivax*) to provide protection from these malaria species or, at least, from severe malaria [35,36]. Due to the presence of several ethnic groups belonging to Arab-Berber or black African ancestry, as well as *P. vivax* [37–39], molecular studies on G6PD and Duffy antigen genotypes in Mauritania may provide insight into how these genotypes/phenotypes interact to determine the most appropriate and safest clinical management of *P. vivax*-infected patients with 8-aminoquinolines.

Recent studies have demonstrated that G6PD deficiency occurs much more frequently in Mauritanians of black African descent [33,40–42]. As for Duffy phenotype or genotype, only very limited epidemiological data are available in Mauritania. In the first known published study on Duffy antigen in Mauritania [43], it was reported that 54% (28/52) of white Moors were Duffy-positive, and 46% (24/52) were Duffy-negative, based on blood group phenotyping. The “black Moors” (defined in that study as those belonging to all other ethnic groups, including Haratins or black Moors, Wolofs, Poulars, and Soninkés) were mostly Duffy-negative (54/55, 98.2%). In another more recent study based on molecular genotyping of blood samples from *P. vivax*-infected patients matched with those who were not malaria-infected [44], most white Moors (186/226, 82.3%) were Duffy-positive, and all cases of *P. vivax* infection (n = 77) were diagnosed exclusively in Duffy-positive white Moors. Among the few febrile Mauritanian patients of black African descent included in that study (n = 14), only two (2/14; 14.3%) were Duffy-positive. Among 12 Duffy-negative patients (12/14, 85.7%), one Duffy-negative patient belonging to Wolof ethnic group was infected with *P. vivax*, supporting the increasing number of recent reports from sub-Saharan African countries that few Duffy-negative Africans can be infected with this malaria species [45–50].

Previous studies conducted in Mauritania have compartmentalized different aspects of human genetics (i.e., Duffy antigens and G6PD activity) and *P. vivax* infection. Intuitively, we hypothesize that Duffy phenotype and G6PD deficiency have an “opposite” effect in Africa [35,36]: non-black Africans are usually Duffy-positive and the African-type G6PD A^–^ genotype is rarely encountered, while most black Africans are Duffy-negative, some of whom may have G6PD A^–^ genotype. Although non-black Africans along the Mediterranean coast may be affected by the Mediterranean-type G6PD B^–^ genotype, we have shown that this genotype is absent in different ethnic groups residing in Mauritania [40]. Based on this hypothesis, Duffy-positive Mauritanians are likely to be white Moors who are less affected with G6PD deficiency, which implies that, in general, they are susceptible to *P. vivax* infection and are likely to have a normal G6PD activity, which allows the administration of the standard dose of primaquine for radical cure. By contrast, Duffy-negative Mauritanians tend to be non-white Moors who are relatively protected from *P. vivax* infection but have a higher probability of being affected by G6PD deficiency. However, in a relatively “rare” case of a Duffy-negative individual being infected with *P. vivax* [44], we may be confronted with the problem of identifying such individuals and prescribing a modified primaquine dose or even withholding anti-hypnozoite treatment with primaquine. In a country like Mauritania where a multitude of peoples of different ethnic origins co-exist, the probability of encountering such a scenario needs to be assessed, and a relevant guideline is required for health practitioners to ensure a safe administration of primaquine. It is in this context that the present epidemiological study was conducted to analyze the allelic frequencies of mutations in Duffy and G6PD genes and predict the possibility of administering 8-aminoquinoline drugs in case of *P. vivax* infection. This scenario extends well beyond the borders of Mauritania and is relevant in other sub-Saharan African countries in continental Africa that have reported *P. vivax* malaria in Duffy-negative patients [45–50]. Other continents, as in Central and South America, where descendants of black African ancestry are present, are also concerned [51–53].

## Materials and methods

### Ethics statement

All patients provided written informed consent to participate in the study, including the use of their blood samples for molecular studies on malaria and genetic studies related to malaria. The study was reviewed and approved by the ethics committee of the Université de Nouakchott Al-Aasriya, Nouakchott, Mauritania (approval no. 112/12-09-2014/USTM, 003/2020/CE/UNA) and the Institutional Review Board of the Institut de Recherche pour le Développement (IRD), Marseille, France (Comité consultative de déontologie et d’éthique approval no. 15/12/2012).

### Study area and human research subjects

Blood samples from patients of both sexes attending different health structures with either symptoms suggestive of malaria (2015–2018) or other unrelated symptoms (2019–2020) were collected after written informed consent. The target populations of these two series of epidemiological studies were (i) febrile patients with symptoms related with malaria (2015–2018) and (ii) non-febrile patients without symptoms suggestive of malaria (2019–2020), respectively. The present study was part of the earlier studies that assessed the prevalence of *G6PD* allelic variants in a large nationwide multicentric patient population (n = 1,101) [33,40]. A subset of archived samples from patients aged > 1 year old (no upper age limit) was selected for studies on G6PD. These samples were also analyzed in earlier studies to detect, identify, and characterize malaria parasites [38,39,54–56].

The patient population was composed of different ethno-linguistic groups representative of the socio-cultural composition of the Mauritanian population. The country’s populations consist of a mosaic of different ethnic groups, including the so-called “white Moors” (Arab and Berber origin, as many peoples in North Africa), the Haratin or sometimes loosely called “black Moors”, and several minority groups of black African origin, namely Wolofs, Poulars, and Soninkés. Due to the government policy, current data on the exact numbers of individuals belonging to different ethnic groups and proportions of ethnic groups in the country are not known.

Samples were collected from five study sites located in different epidemiological strata that define the geographic distribution of malaria in the country [57]: Atar (an oasis city in northern Saharan zone), Nouakchott (the capital city located in the Saharan zone along the Atlantic coast), Aleg (southwestern Sahelian zone), Rosso (Sahelian zone along the Senegal River basin and on the border with Senegal), and Kobeni (Sahelian-Saharan transition zone near the frontier with Mali). The map showing these study sites can be found in the earlier published work [40]. The prevalence of *P. falciparum* and *P. vivax* malaria among febrile patients in four of five above-mentioned sites was published elsewhere (S1 Table). Based on our field studies on malaria and/or G6PD deficiency conducted in different regions of the country [33,37–40,55,56], it has been observed among the patients consulting health centers and hospitals that white Moors are largely predominant in Atar whereas various proportions of ethnic groups make up the populations living in the other four study sites. The detailed distribution of patient populations in terms of their ethnic origin is described in our earlier works [33,40].

### DNA extraction

DNA was extracted from dried blood spots based on the protocol described in our earlier work [40]. Briefly, two to three drops (100–150 µL) of fingerpick capillary blood were imbibed onto Whatman grade 3MM filter paper (GE Healthcare UK Ltd., Little Chalfont, Buckinghamshire, UK) or Whatman FTA card (GE Healthcare), air dried, and stored in a sealed plastic sachet with a desiccant at −20 °C. Filter papers and cards impregnated with blood were sent to France at ambient temperature and stored at −20 °C until DNA extraction. A 1 mm-diameter disc of filter paper or card imbibed with dried blood was punched out and placed in 96-well plates. Genomic DNA was extracted from blood specimens using an automated MagMAX-Express system (Thermo Fisher Scientific, Montigny-le-Bretonneux, France) according to the manufacturer’s instructions.

### G6PD genotyping

The key *G6PD* mutations that occur in northern (i.e., Mediterranean-type) and sub-Saharan Africa (i.e., type A^–^) were determined by PCR-restriction fragment length polymorphism (RFLP) and sequencing and published in our earlier studies [33,40]. *G6PD* sequence data published in those studies are referred in the present study to analyze the relationship between *G6PD* and Duffy antigen genotypes. Based on the nucleotide sequences of positions 202, 376, 542, 680, and 968, G6PD genotypes were designated B in hemizygous normal males and BB in homozygous normal females (normal haplotype GAACGT; phenotype G6PD B), based on the 1985 WHO classification of G6PD deficiency [34,35]. In the presence of a single A376G mutation, the phenotype is designated G6PD A, which has the same enzymatic activity as G6PD B. Its corresponding genotypes are A in hemizygous males and AA or AB in females. In Africa, G6PD deficiency is generally associated with either the African-type G6PD A^–^or Mediterranean-type G6PD B^–^ variants. The latter was absent in our samples. The African-type G6PD A^–^ genotypes are designated A^–^ in hemizygous enzyme-deficient male (haplotypes **AG**ACGT, G**GT**CGT, **GG**AC**T**T, or **GG**ACG**C**), A^–^A^–^ in homozygous enzyme-deficient females, and AA^–^ or BA^–^ in heterozygous females. The African-type G6PD A^–^ occurs in the presence of two mutations, one of which is A376G. Unless otherwise stated, the present work refers to earlier studies conducted in Mauritania based on the 1985 WHO classification of G6PD deficiency [34,35].

### High-throughput mutation analysis using multiplex amplicon-based DNA sequencing

Pooled next-generation sequencing (NGS) with barcoding is an efficient method for genotyping target genes in a large number of samples [58]. More recently, targeted amplicon sequencing has been developed as an even more cost-effective, high-throughput versatile approach that targets specific polymorphisms to characterize a panel of multiple target single nucleotide polymorphism (SNP) markers and has been shown to meet a wide range of genetic applications [59–61]. Based on these recent technological advances in genome sequencing, a novel multiplex amplicon-based method was designed and developed in the present study to determine Duffy blood group genotype. This approach allows a simultaneous characterization of the polymorphisms that occur between nucleotide positions T-69C and G408A. Duffy phenotype was predicted on the basis of the currently accepted nomenclature [62,63].

Two non-overlapping regions of the atypical chemokine receptor 1 (*ACKR1*) gene, which encodes DARC (also referred to as Fy glycoprotein or sometimes as the cluster of differentiation 234 [CD234]), were amplified by polymerase chain reaction (PCR): a region flanking exon 1 that spans the promoter region (392 base pair [bp] fragment; from nucleotide 5,760–6,151 of NG_011626.3 RefSeq gene) and a second region flanking the 5’-end of exon 2 (541 bp; from nucleotide 6,327–6,867 of NG_011626.3 RefSeq gene). Both targeted genomic regions were amplified in a single reaction to generate sufficient amount of templates for subsequent high-throughput sequencing. Multiplex PCR amplifications were performed with 5 μL of purified DNA template in a 20 μL reaction mixture composed of 5 μL of Hot START 5 × BIOamp DNA polymerase mix (Microsynth France, Lyon, France), 1 μL of forward and reverse primers (10 μM; a total of 4 μL for four primers), and 11 μL of water. The primers used in this study are shown in S2 Table. The thermal cycler was programmed as follows: initial activation of polymerase at 96°C for 10 min, followed by 35 cycles of (i) denaturation at 96°C for 30 sec, (ii) hybridization at 62°C for 30 sec, and (iii) extension at 72°C for 1 min, followed by a final extension step at 72°C for 7 min to complete the synthesis of all PCR products.

Illumina Nextera universal tail sequences were added to the 5’-end of each of these primers to facilitate the preparation of the library by a two-step PCR approach. A barcode was inserted in the sequences of the forward primers (S2 Table). The multiplex protocol involved the use of the same barcode on each column of a 96-well plate so that 10 μL of amplified products could be pooled per lane (i.e., 12 samples were pooled into a single tube with a final volume of 120 μL). This multiplexing scheme allowed a 12-fold reduction in the number of samples to be sequenced, i.e., 96 samples from one 96-well plate were pooled and grouped into eight different tubes, equivalent to a column of a 96-well plate. The pooled amplicons were purified using a 0.8 × magnetic beads (SPRIselect beads, Beckman Coulter France SAS, Roissy CDG, France). Fifteen cycles of PCR amplification were performed using Nextera Index Kit – PCR primers (Illumina France, Evry, France), according to the manufacturer’s instructions, to add the P5 and P7 termini that bind to the flow cell and the dual 8 bp index tags. Barcoded and indexed samples were pooled and quantified by fluorometric method (QuantiFluor dsDNA System; Promega France, Charbonnières-les-Bains, France) and visualized on QIAxcel Capillary Electrophoresis System (Qiagen France, Les Ulis, France). Libraries were sequenced on a MiSeq run (Illumina France) using MiSeq v3 chemistry with 300 bp paired-end sequencing (Microsynth France - Biofidal, Vaulx-en-Velin, France).

The DDemux demultiplexer software program was used to demultiplex fastq files according to the P1 barcodes inserted at the 5’-end of each sequence [64]. After demultiplexing, trimmomatic v0.33 was used to discard reads shorter than 32 nucleotides, filter out Illumina adaptor sequences, remove leading and trailing low-quality bases, and trim reads when the average quality per base dropped below 15 on a 4-base-wide sliding window [65]. The trimmed reads were aligned to the NG_011626.3 RefSeq gene sequence with bowtie2 v.2.1.0 [66]. The alignment file was converted, sorted, and indexed using Samtools v0.1.19 [67–69]. Coverage and sequencing depth were assessed using bedtools v2.17.0 [70]. DNA variants were called using mpileup from bcftools v1.9 using a maximum coverage per locus of 10,000 instead of the default 250 to consider the high depth of amplicon sequencing [71]. Variant calling files were concatenated into a single tab-delimited file that included the variants from all patients. The individual genotypes based on 11 major mutations previously reported to be associated with Duffy blood group system expression and its isoforms were retrieved from the variant calling files using an awk command (S1 File) [72]. Nucleotide positions with a mapping quality < 60 and a sequencing depth < 10 were discarded from further analysis.

### Statistical analysis and data visualization

For G6PD genotyping, archived blood samples were randomly selected from the first series of epidemiological studies on malaria prevalence, with the exception of samples from Kobeni where a large number of samples (> 2,000) were available. In Kobeni, 327 samples were selected based on two criteria: the absence of malaria parasites and preference for patients belonging to one of the minority groups of black African origin.

Descriptive statistics and data visualization were performed in the statistical environment R (S2 File) [73]. Figures were made using the package ggplot2, ggstatsplot, Tidyverse environment [74–76], and GraphPad Prism v5 (GraphPad Software, Boston, MA). Pearson’s chi-squared test with simulated p-value (based on 2000 replicates) was used to analyze contingency tables to compare the distribution of Duffy genotypes and predicted phenotypes and the distribution of G6PD deficiency and Duffy phenotypes. Fisher’s exact test was used to assess whether Duffy phenotypes are associated with ethnic origins.

## Results

### G6PD polymorphism according to sex and ethnic groups

Data on *G6PD* genotypes (samples collected in 2015–2018) were published elsewhere [40]. Briefly, 3.8% (19/499) of white Moors (10 males [202A, n = 6; 542T, n = 2; 968C, n = 2], 3 homozygous females [202A/A, n = 2; 968C/C, n = 1], and 6 heterozygous [202G/A, n = 5; 542A/T, n = 1] females) carried the African-type *G6PD* A^–^ variant generally associated with mild-to-moderate G6PD deficiency. There were 12.3% (39/316) of black Moors (15 males [202A, n = 12; 968C, n = 3], 3 homozygous females [202A/A, n = 1; 968C/C, n = 2], and 21 heterozygous females [202G/A, n = 14; 542A/T, n = 1; 968T/C, n = 6]) with African-type *G6PD* A^–^ variant. Among those belonging to the other ethnic groups of black African descent, i.e., Wolofs, Poulars, and Soninkés, 10.5% (19/181; 3 males [202A, n = 1; 968C, n = 2], 2 homozygous females [968C/C, n = 2], and 13 heterozygous [202G/A, n = 4; 968T/C, n = 9] females; 1 missing information on sex) were affected with *G6PD* A^–^ variant. None of the included individuals carried the Mediterranean-type *G6PD* B^–^ genotype, which is often associated with severe G6PD deficiency.

Blood samples from 323 additional patients (74 males and 249 females) included in a follow-up study conducted in Mauritania in 2019–2020 were also analyzed [33]. In this second study, 3.5% (5/142) of white Moors (1/45 males, 4/97 BA^–^ heterozygous females, no homozygous female) were characterized to be *G6PD* A^–^. There were 18.9% (14/74) of black Moors (3/18 males; of 56 females, 1 homozygous and 10 BA^–^ or AA^–^ heterozygous) with African-type *G6PD* A^–^ variant. Among those belonging to the other ethnic groups of black African descent, including those who were of mixed ethnic descent, 15.5% (16/103; 1/9 male; 15/94 females, 1 homozygous and 14 BA^–^ or AA^–^ heterozygous; missing information on ethnic origin in 2 males and 2 females) were affected with *G6PD* A^–^ variant. The Mediterranean-type *G6PD* B^–^ genotype was not observed.

### Duffy blood group genotypes and predicted phenotypes

**Fig 1:**
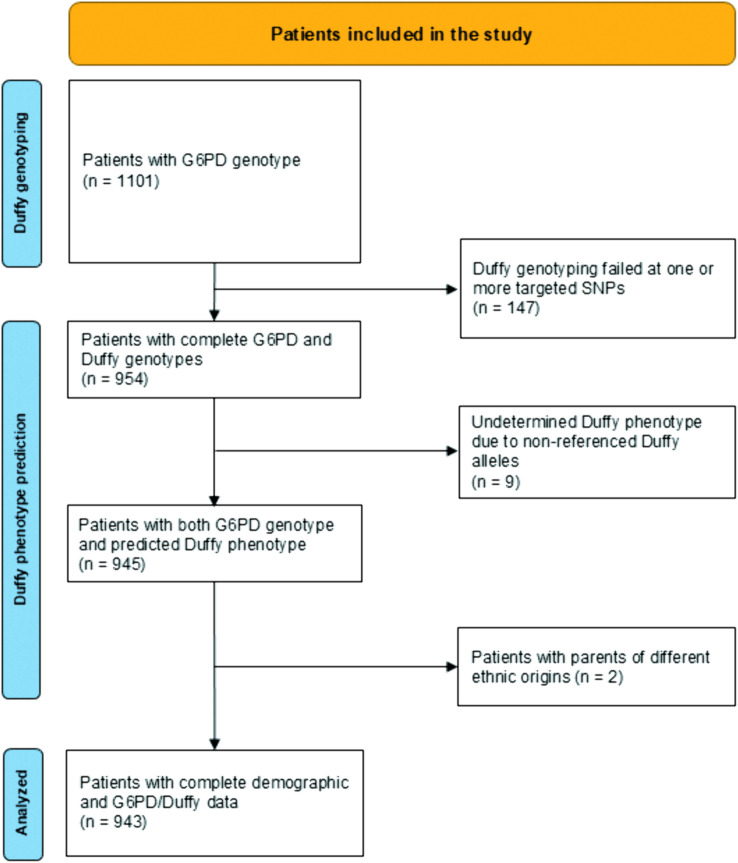
Flow chart ([77]).

Genotypes of Duffy blood group system were determined by multiplex amplicon-based sequencing in a total of 1,101 blood samples (427 males and 674 females) (Fig 1). Interpretable sequence data on Duffy antigen polymorphisms at all 11 codons (or specific nucleotides in the promoter region) were available from 954 (86.6%) samples. PCR amplification failed either partially at one or several targeted SNPs or completely for all 11 SNPs in 147 (13.4%) samples most likely due to an inadequate amount of blood spots and/or possible DNA degradation associated with poor conservation conditions before transporting the samples from the field to the laboratory in France (note: the archived samples were collected in 2015–2020 and exposed to high ambient temperature in Mauritania). Because of the limited amount of blood spots available for each sample, no attempt was made to repeat PCR sequencing in case of failure. A similar PCR success rate was found using the same samples in an earlier study on *G6PD* genotyping [40]. Among 954 successfully sequenced samples with complete results at 11 SNP markers, the mean sequencing depth across individual samples was 7000 X (25^th^ percentile, 485 X; 75th percentile, 9060 X) for the amplicon harboring the promoter region, and 652 X (25^th^ percentile, 36 X; 75^th^ percentile, 808 X) for the second amplicon flanking the 5’-terminus of the exon 2 (S1 Fig).

Eleven major mutations known to be associated with Duffy blood group system and its isoforms were identified in blood samples obtained from Mauritanian patients (Table 1). Fixed wild-type alleles were observed in all patients at seven SNPs: T-69C, G145T, G266A, G287A, G395A, G407A, and G408A. Co-dominant FY*01 and FY*02 alleles (also commonly referred to as the *FYA* and *FYB* alleles, respectively), which differ by a SNP at nucleotide 125, were used to define the phenotypes, based on the criteria presented in Table 1.

**Table 1:** Mutations in the atypical chemokine receptor 1 gene (ACKR1 gene coding for the Duffy blood group system) analyzed by multiplex amplicon-based DNA sequencing.

Allele name	Nucleotide change	Phenotype	Amino acid substitution	Gene region	Position
FY*01	125G	Fy(a+)	No	Exon 2	6552
FY*02	125G > A	Fy(b+)	Gly42Asp	Exon 2	6552
Weak FY*01 phenotypes					
FY*01W.01	265C > T	Fy(a + ^w^)	Arg89Cys	Exon 2	6692
FY*01W.02	298G > A	Fy(a + ^w^)	Ala100Thr	Exon 2	6725
Weak FY*02 phenotypes					
FY*02W.01	265C > T 298G > A	Fy(b + ^w^)	Arg89Cys Ala100Thr	Exon 2	6692 6725
FY*02W.02	145G > T	Fy(b + ^w^)	Ala49Ser	Exon 2	6572
FY*02W.03	266G > A	Fy(b + ^w^)	Arg89His	Exon 2	6693
Null phenotypes, FY*01 alleles					
FY*01N.01	-67T > C	Fy(a^–^b^–^)	No	5’-UTR	5881
FY*02N.02	407G > A	Fy(a^–^b^–^)	Trp136Ter	Exon 2	6834
FY*01N.03	408G > A	Fy(a^–^b^–^)	Trp136Ter	Exon 2	6835
FY*01N.04	287G > A	Fy(a^–^b^–^)	Trp96Ter	Exon 2	6714
FY*01N.06	395G > A	Fy(a^–^b^–^)	Gly132Asp	Exon 2	6822
FY*01N.08	-69T > C	Fy(a^–^b^–)^	No	5’-UTR	5879
Null phenotypes, FY*02 alleles					
FY02*N.01	-67T > C 125G > A	Fy(a^–^b^–^)	No Gly42Asp	5’-UTR Exon 2	5881 6552

The present study was designed to identify 11 major atypical chemokine receptor 1 (ACKR1; also referred to as FY and DARC) gene mutations. Their predicted Duffy phenotype and the current Duffy blood group nomenclature were based on earlier works [62,63]. The phenotype described here refers to the erythroid cells. The weak phenotypic expression of either FY*A or FY*B allele is denoted by the letter “w.” FY*01 allele is also referred to as FY*A allele; FY*02 allele is also called FY*B allele. These alleles differ by a single nucleotide at position 125. The gene sequence consists of two exons. 5’-untranslated region (5’-UTR) refers to the GATA promoter region of the FY*B allele. Specific mutations in the promoter region lead to the disruption of the binding site for the GATA-1 erythroid transcription factor, which in turn leads to the absence of Duffy antigen expression on the erythrocyte surface; non-erythroid cells are not affected. In addition to this major genetic mechanism underlying Duffy-negative phenotype, some non-synonymous mutations occurring in exon 2, as illustrated in this table, may also result in Duffy null “erythrocyte silent” (ES) phenotype. “Position” denotes the nucleotide number in the reference gene NG_011626.3 where the mutation occurs.

Nineteen individuals (19/954; 1.9%) were homozygous for the FY*01 allele, and 681 individuals (681/954; 71.4%) were homozygous for the FY*02 allele (Table 2). Among these 681 individuals homozygous for the FY*02 allele, 165 (17.3%) carried the T > C mutation at nucleotide position T-67C which prevents Fyb antigen expression only in the red blood cells in one of the chromosome pairs (*i.e.*, genotype FY*02/FY*02N.01 and phenotype Fy(a + b^–^)) and 430 (45.1%) in both chromosome pairs (*i.e.,* genotype FY*02N.01/FY*02N.01 with a Duffy null or erythrocyte silent (ES) phenotype Fy(a^–^b^–^)).

**Table 2:** Duffy antigen genotypes and predicted phenotypes in Mauritanian patients.

Genotypes	Phenotypes	Number of patients (%)
Homozygous		
FY*01/FY*01 (or *FYA/FYA*)	Fy(a + b^–^)	19 (2.0)
FY*02/FY*02 (or *FYB/FYB*)	Fy(a^–^b+)	84 (8.8)
FY*02/FY*02N.01	Fy(a + b^–^)	165 (17.3)
FY*02N.01/FY*02N.01	Fy(a^–^b^–^)	430 (45.1)
FY*02/FY*02W.01	Fy(b + ^w^)^1^	2 (0.2)
Total homozygotes		700 (73.4)
Heterozygous		
FY*01/FY*02	Fy(a + b+)	116 (12.2)
FY*01/FY*02N.01	Fy(a + b^–^)	119 (12.5)
FY*01N.01/FY*02N.01	Fy(a^–^b^–^)	10 (1.0)
FY*02/non-referenced allele ^**2**^	Undetermined	9 (0.9)
Total heterozygotes		254 (26.6)

The total number of successfully sequenced samples, defined as the samples for which all 11 SNPs selected and analyzed in the present study were successfully determined, was 954. Among these samples, 700 were homozygous, and 254 were heterozygous. The denominator to calculate the percentages in this table is 954. The phenotype Fy(a^–^b^–^) corresponds to Duffy-negative phenotype. G407A mutation, which leads to Duffy-negative phenotype, was not found in any of the samples. Nine individuals were carriers of non-referenced G125C (n = 5) or G125T nucleotide change.

^1^ Although FY*02W.01 allele is associated with a weak expression of Fyb antigen, the presence of the other allele (FY*02) in these patients is predicted to compensate for the weak expression, resulting in a normal expression.

^2^ “Non-referenced allele” refers to either G125C or G125T.

A total of 254 (254/954, 26.6%) individuals were heterozygous for the FY*01 and FY*02 alleles, among whom 116 (12.2%) were predicted to express both Fya and Fyb antigens normally (*i.e.*, genotype FY*01/FY*02 and phenotype Fy(a + b+)). A total of 119 (12.5%) carried the FY*02 allele with the T > C mutation at nucleotide position T-67C which prevents Fyb antigen expression (*i.e.*, genotype FY*01/FY*02N.01 and phenotype Fy(a + b^–^)). Ten patients (1%) had FY*01N.01/FY*02N.01 genotype, which corresponds to a Duffy null phenotype Fy(a^–^b^–^) (Table 2).

Two other mutations were reported to prevent Fyb antigen expression: G407A and G781A. The nucleotide G407 was wild-type in all samples. The amplicon-based method used in the present study was not designed to determine the SNP at nucleotide 781 in exon 2.

The weak expression of both Fya and Fyb antigens was determined by the co-occurrence of mutations C265T and G298A (genotype FY*02W.01). Only two individuals (0.2% of all samples) carried the FY*02W.01 (C265) allele. These two individuals were, however, not predicted to have a weak Fyb antigen expression because they also carried a FY*02 allele (*i.e.*, genotype FY*02/FY*02W.01) [78]. All other alleles known to be associated with the weak expression of Fya or Fyb antigen (*i.e.*, G145T and G266A) were either fixed in all samples or not included in the present study (*i.e.*, C901T).

Five patients carried a non-referenced G > C mutation at position 125 at the heterozygous state with allele FY*02. Of these five cases, four were homozygous C at position T-67C, which would probably result in Duffy null phenotype Fy(a^–^b^–^), while one patient carried both T and C nucleotides at this position. Four additional individuals carried a non-referenced G > T mutation at position 125 at the heterozygous state with allele FY*02, two of whom had the T > C mutation at nucleotide position -67 at the homozygous state. These mutations are non-synonymous and encode an alanine (G125C) or a valine (G125T). We did not predict the Duffy blood group phenotypes for these individuals, including those with missing genotypes at nucleotide positions 125 and/or -67.

**Fig 2:**
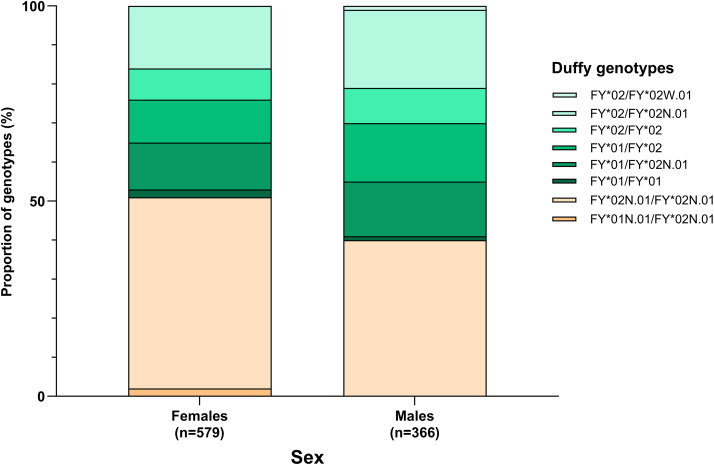
Proportions of different Duffy genotypes with respect to sex. Duffy genotypes in green and orange correspond to Duffy phenotype + and –, respectively.

**Fig 3:**
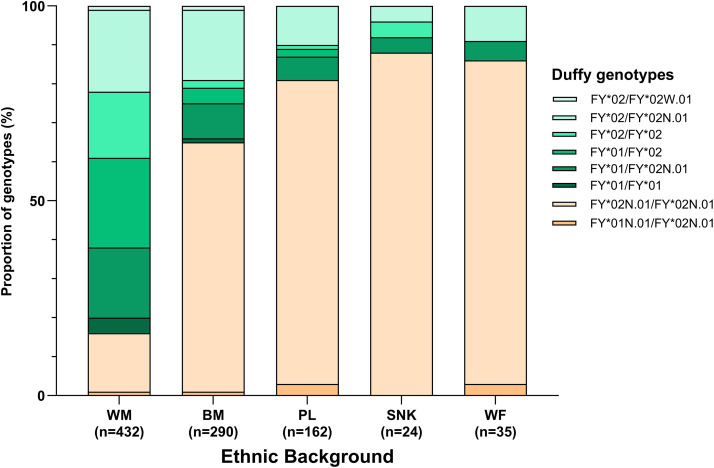
Proportions of different Duffy genotypes with respect to ethnic background. Duffy genotypes in green and orange correspond to Duffy phenotype + and –, respectively. Individuals who declared a double ethnic origin (n = 2) were excluded from these figures. Ethnic background: Black Moors (BM), White Moors (WM), Pular (PL), Wolof (WF), and Soninké (SNK).

Duffy genotypes were not equally distributed between the sexes and among ethnic groups (Figs 2 and 3). The predicted Duffy blood phenotypes were significantly associated with sex, with females (295/579, 50.9%) being more affected with Duffy-negative phenotype than in males (145/366, 39.6%) (Pearson’s chi-squared test with simulated *p*-value, p = 0.001) (Fig 2). This unexpected, slightly skewed distribution of Duffy genotypes between sexes was probably due to sampling bias in our hospital-based studies which included patients enrolled in the Mother and Child Hospital. The genotype FY*01N.01/FY*02N.01 was exclusively represented in 10 females, while FY*02W.01 allele was only found in two males.

Genotypes conferring a non-expression of the Duffy antigen on red blood cells (*i.e.*, Duffy null phenotype associated with the genotypes FY*01N.01/FY*02N.01 and FY*02N.01/FY*02N.01) were overrepresented in individuals of Black African descent (ranging from 65% to 88% in Black Moors, Pular, Wolof, and Soninke, as compared to 16% in White Moors) (Fig 3). The predicted Duffy blood phenotypes were significantly associated with ethnic origins (Fisher’s exact test with Monte Carlo p-values simulation, p < 0.001).

### Predicted Duffy blood group expression and G6PD deficiency in different ethnic groups

Overall, G6PD-deficient genotypes patients represented 9.8% of cases included in the present study (n = 943), with 16.6%, 5.6%, 2.9%, and 1.7% of individuals with Fy(a^–^b^–^), Fy(a^–^b+), Fy(a + b^–^), and Fy(a + b+) phenotypes, respectively (Table 3).

**Table 3:** Relationship between *G6PD* genotypes and predicted Duffy phenotypes in Mauritanians.

G6PD/Duffy	Sex	Number of patients belonging to different ethnic groups					Total number (%)
		WM	BM	PL	SNK	WF	
G6PD A^–^	F	0	3	1	1	1	6 (0.6)
	M	2	11	3	0	1	17 (1.8)
G6PD B	F	34	99	70	16	20	239 (25.3)
	M	26	54	37	3	7	127 (13.5)
Heterozygous	F	6	22	20	1	1	50 (5.3)
**Fy(a**^**–**^**b**^**–**^)							***439 (46.6)***
G6PD A^–^	F	1	0	0	0	0	1 (0.1)
	M	5	3	0	0	0	8 (0.8)
G6PD B	F	89	26	14	1	3	133 (14.1)
	M	71	28	4	0	0	103 (10.9)
Heterozygous	F	0	3	1	1	0	5 (0.5)
**Fy(a** ^**–**^ **b+)**							***250 (26.5)***
G6PD A^–^	F	0	1	0	0	0	1 (0.1)
	M	1	0	0	0	0	1 (0.1)
G6PD B	F	54	15	7	1	2	79 (8.4)
	M	42	13	0	0	0	55 (5.8)
Heterozygous	F	0	0	2	0	0	2 (0.2)
**Fy(a + b**^**–**^)							***138 (14.6)***
G6PD A^–^	F	0	0	0	0	0	0 (0)
	M	0	1	0	0	0	1 (0.1)
G6PD B	F	55	6	0	0	0	61 (6.5)
	M	46	5	2	0	0	53 (5.6)
Heterozygous	F	0	0	1	0	0	1 (0.1)
**Fy(a + b+)**							***116 (12.3)***
**Total number**		**432 (45.8)**	**290 (30.8)**	**162 (17.2)**	**24 (2.5)**	**35 (3.7)**	**943 (100)**

G6PD A^**–**^ denotes African-type G6PD deficiency, usually associated with mild to moderate G6PD enzyme activity. 8-aminoquinolines may be administered with caution to patients with G6PD A^**–**^ deficiency, usually at a modified weekly dose over 8 weeks instead of the standard 14-day treatment. “G6PD B” denotes normal G6PD activity. “G6PD B^**–**^” refers to the Mediterranean-type G6PD deficiency, often associated with severe signs and symptoms when the affected individual encounters a triggering factor (infection, consumption of fava beans, intake of certain medications, including 8-aminoquinolines). None of the patients included in the study was affected with the Mediterranean-type G6PD deficiency. The variants are based on the 1985 WHO classification of G6PD deficiency [34,35]. The gene encoding G6PD is on chromosome X. Males are hemizygous (B for normal enzyme activity without any mutation, A for normal enzyme activity with a single A376G mutation, or A^**–**^ for enzyme deficiency due to the presence of the A376G mutation + a second mutation at nucleotide positions 202, 376, 542, 680, or 968); females may be homozygous (BB for normal enzyme activity without any mutation, AA for normal enzyme activity with a single A376G mutation in both alleles, A^**–**^A^**–**^) or heterozygous (BA for normal enzyme activity; AA^**–**^ or BA^**–**^, the enzyme activity cannot be predicted in heterozygous females). Individuals with a declared double ethnicity were excluded. Ethnic background: Black Moors (BM), White Moors (WM), Pular (PL), Wolof (WF) and Soninke (SNK).

Analysis of G6PD deficiency genotypes by sex and ethnic groups did not show any statistically significant association with Duffy phenotypes (chi-squared test, p > 0.1). Among 432 white Moors with both Duffy and *G6PD* genotype data, 356 (82.4%) were Duffy-positive, of whom only 7 (2.0%) carried the African-type *G6PD* A^–^ mutations. Of the remaining 76 (17.6%) white Moors, 8 (10.5%) were Duffy-negative and affected with G6PD A^–^ type deficiency, mostly in heterozygous females (n = 6). Of 511 individuals of black African ancestry with both Duffy and *G6PD* genotype data, 140 (27.4%) were predicted to be Duffy-positive, i.e., Fy(a^**–**^b+), Fy(a + b^**–**^), or Fy(a + b+), and 13 (9.3%, including heterozygous females) were carriers of mutations associated with African-type G6PD A^–^ deficiency. Among Duffy-negative Fy(a^**–**^b^**–**^) individuals of black African ancestry, 65/371 (17.5%) were affected by African-type G6PD A^–^ deficiency, mostly (n = 44) in heterozygous females.

## Discussion

The updated epidemiological data on Duffy antigen in Mauritanian populations confirm our initial hypothesis that white Moors, who are descendants of Arab and Berber ethnic groups genetically and historically related to peoples in North Africa, are predominantly Duffy-positive (84%). In a study conducted by genotyping (PCR sequencing by Sanger method) in 2007–2009 exclusively in samples collected in Nouakchott, a closely similar proportion of Duffy-positive white Moors (82.3%; total number of white Moors included, 221) was reported [44]. By contrast, another earlier study based on blood group phenotyping in Nouakchott in 1984 reported a much lower proportion of Duffy-positive white Moors in a small sample size (54%, n = 52) [43].

Mauritania straddles between the Maghreb region to the north and the Sahel to the south, and a large part of its territory is occupied by the Sahara desert. Global mapping of the distribution of FY*B allele generated from modeling has suggested that at the periphery of the region where Fy*B^*ES*^ allele predominates, as in sub-Saharan Africa, Fy*B tends to be prevalent [79]. Although this predicted model tends to agree with the results obtained in the present study, 16% of white Moors were Duffy-negative. This is a relatively high proportion of Duffy-negative phenotype in an ethnic group that is supposedly “homogeneous” and may suggest the limits of modeling at the country-level without evidence-based data and historical considerations. The populations in Africa have undergone various interactions and migrations over the past centuries, resulting in a complex human genetic structure, but the extent of population movement along the east-west and north-south axes within the African continent and also into Africa (e.g., from the current Middle East), as well as the roles played by autochthonous peoples, consanguinity, and centuries of Arab slave trade, is still being hotly debated [80–83]. Nonetheless, the cultural and linguistic ties and geographic proximity to the Maghreb are obvious factors to be taken into consideration to explain our observations concerning white Moors. For example, in Algeria, a neighboring country to the northeast of Mauritania, in sharp contrast to the peoples of Arab descent (8.9–10.4% Duffy-negative), a Berber population residing in central region of Algeria has been characterized as predominantly (i.e., 62%) Duffy-negative [84]. Similarly, in Rabat and several cities in south-central Morocco where both populations of Arab and Berber origin co-exist, the prevalence of Duffy-negative subjects, as determined by either phenotyping or genotyping, ranged from 11.1% to 24.8% [85,86]. In Tunisia, where a minority Berber ethnic group also exists, it was reported that 11.3% (13/115) of the general population is Duffy-negative [87]. It has been advanced as an hypothesis that genotypes associated with Duffy-negative phenotype in peoples of Arab descent may have originated, at least in part, from the Middle East, where high prevalence of Duffy-negative phenotype (61–78%) has been reported, instead from sub-Saharan Africa [84,88,89]. This hypothesis is supported by the historical fact regarding the Arab-Islamic conquest of the Maghreb during the seventh and eighth centuries A.D.

Data on Duffy genotypes generated in the present study need to be confronted with *G6PD* genotypes which were also evaluated for the same samples [40]. Only nine of 423 (2.1%) white Moors were characterized to have the African-type *G6PD* A^–^ genotype, and only two of them (0.5%), both in males, were also Duffy-negative. The source of African-type *G6PD* A^–^ genotype in white Moors living in Mauritania has not been elucidated. Although it can reasonably be argued that the African-type *G6PD* A^–^ genotype most likely arrived to Mauritania with peoples originating from sub-Saharan Africa, an alternative hypothesis that it was derived through persons in the Maghreb and in the Middle East cannot be totally refuted at present. For example, in Algeria and Morocco, two neighboring countries of Mauritania to the north, both the Mediterranean-type G6PD B^–^ and African-type G6PD A^–^ deficiencies co-exist in the patient population with clinical symptoms (anemia, favism, neonatal jaundice), with about 50% of the cases being due to the African-type G6PD A^–^ deficiency [90,91]. In Arab countries in the Middle East, the Mediterranean-type G6PD B^–^ deficiency predominates over other types of G6PD deficiencies, but in a small minority of G6PD deficient Arab patients (generally < 2%, similar to the proportion found in Mauritanian white Moors), the deficiency is associated with the African-type G6PD A^–^ [92–94]. However, the fact that the Mediterranean-type G6PD B^–^ deficiency has not been found in Mauritania argues against the hypothetical north-to-south (i.e., from the Maghreb) and east-to-west (i.e., from the Middle East) genetic flow and seems to favor the south-to-north axis, i.e., from sub-Saharan Africa. Further surveillance of Mediterranean-type G6PD B^–^ deficiency in Mauritania and comparative genome analysis of other genes would be required for a more conclusive evidence supporting the latter hypothesis on gene flow.

The available epidemiological data on malaria in Mauritania have indicated that *P. vivax* malaria is largely a disease that affects white Moors [38,39,44]. These data are in line with the findings that the presence of Duffy antigen on reticulocyte surface is one of the major requirements for *P. vivax* infection. Since 2014, the Mauritanian Ministry of Health recommends ACT and primaquine to treat *P. vivax* infection [95]. The results of the present study suggest not only that a large majority of white Moors are Duffy-positive and susceptible to *P. vivax* infection, but also that they are eligible for anti-hypnozoite therapy with primaquine at the standard dose. It would not be probably cost-effective to systematically screen for G6PD activity in *P. vivax*-infected white Moors, as in northern Mauritania where they are largely predominant. In the rare case when gross hematuria or other signs of hemolytic anemia are encountered by the white Moor patient within the first three days of primaquine treatment due to mild to moderate G6PD deficiency associated with G6PD A^–^ genotype (class B according to the 2024 revised WHO classification of G6PD variants) [96], drug treatment can be immediately suspended while awaiting for further laboratory examinations to adapt the treatment. The question on whether Duffy-negative white Moors are relatively more protected from *P. vivax* infection than Duffy-positive white Moors has not been addressed in our studies.

As for individuals belonging to ethnic groups of black African descent, who are genetically and culturally related to populations living in sub-Saharan Africa, the present study confirmed our hypothesis that a large majority of them (65–88%, depending on the ethnic background) are Duffy-negative. A previous molecular study conducted in Mauritania demonstrated that *P. vivax* infection occurs rarely in these populations, reinforcing the argument that Duffy antigen remains the major receptor and pathway for parasite invasion into reticulocytes [44]. Despite the mounting evidence of the occurrence of *P. vivax* malaria in Duffy-negative African patients elsewhere in the African continent [45–50], this phenomenon appears to be more of an exception, rather than the rule, at least in Mauritania at present. Moreover, it remains unknown whether the parasite invades reticulocytes through alternative pathways [97,98]. A recent experimental study has reported that *P. vivax* can invade Duffy-negative erythroblasts, some of which express functional DARC transiently during erythropoiesis (i.e., the mean of 1.0–3.2% of Duffy-negative erythroblasts between day 0 and day 12, compared to 24.6–78.3% in Duffy-positive erythroblasts) [99]. If the results of these experiments are confirmed, it can be deduced that alternative pathways are not necessary to explain the widespread presence of *P. vivax* in sub-Saharan Africa. These experimental findings are also in agreement with the clinical observation that *P. vivax* occurs at low parasitemia due, not only to the low number of circulating reticulocytes under physiological conditions in the human host, but also to the low number of erythroblasts which transiently express DARC in Duffy-negative individuals [99].

About one-fifth (65/337; 19.3%) of Duffy-negative individuals of black African ancestry were affected by G6PD deficiency, including heterozygous females whose G6PD phenotype cannot be predicted with accuracy from the genotype due to random X chromosome inactivation unless the enzymatic activity is measured by spectrophotometry or an alternative, reliable field-compatible tool [31,33,100,101]. However, these Duffy-negative, G6PD-deficient individuals are likely to be relatively protected from *P. vivax* malaria and are expected to require primaquine therapy only on rare occasions. The patients who may potentially encounter a problem with primaquine treatment for *P. vivax* infection are those who are both Duffy-positive and G6PD-deficient. Our results suggested that 7 of 432 (1.6%) white Moors and 13 of 511 (2.5%) Mauritanians of black African ancestry, i.e., a total of 20 of 943 patients (2.1% of our patient population, including both sexes), fall into this at-risk category. Faced with a laboratory-confirmed diagnosis of *P. vivax*, systematic screening and determination of the level of G6PD enzymatic activity are required before administering primaquine for ethical and safety reasons [2,29–33].

In addition to Duffy antigen and G6PD, the hepatic cytochrome P-450 isozyme 2D6 (CYP2D6) plays a crucial role in the expected efficacy of anti-relapse therapy. Primaquine is a pro-drug which requires hepatic transformation to its active metabolite, hydroxy-primaquine. Some CYP2D6 gene polymorphisms result in null or impaired drug metabolism, leading to low plasma concentration of the active metabolite and failure to prevent relapse in “poor metabolizers” [102–105].

About 40% of the population exposed to the risk of *P. vivax* infection in southeast Asia, where relevant data are available, is not eligible for the standard anti-relapse treatment with primaquine due to G6PD deficiency and may face treatment failure due to poor drug metabolism [103]. There are no data on *CYP2D6* polymorphisms in Mauritanian populations. Data in the literature suggest that the lowest proportion of populations with a normal CYP2D6 activity is found in East/Southeast Asia and Africa [106]. Among the studied populations, 56% in West Africa (49–60% in sub-Saharan Africa) and 32% in North Africa (30–32% in Algeria and Morocco, two neighboring countries to the north of Mauritania) were characterized to be either null or impaired metabolizers, of whom 15–21% of the individuals in West and North Africa (15% in Algeria and 21% in Morocco) had null metabolism [106–109]. If we assume that Mauritanian white Moors are genetically closely related to Algerian and Moroccan populations, about 69% of them are expected to have a normal CYP2D6 activity. Based on this assumption and our data on Duffy and G6PD, it can be predicted that, after G6PD screening is performed, the proportion of *P. vivax*-infected white Moor patients eligible for the standard anti-hypnozoite primaquine therapy is 56.6% in males and 56.8% in females. Our data are in agreement with the earlier prediction made in Asian populations that about 40% of the population potentially exposed to the risk of *P. vivax* infection (43% of male and female white Moors) would not benefit from the standard anti-relapse treatment with primaquine due, in part, to G6PD deficiency and, more importantly, to poor drug metabolism [103].

Several limitations of the present study should be noted. First, only a limited number of mutant alleles were assessed. Duffy genotypes are highly complex [62,63], and many “minor” mutations in the *ACKR1* gene that can occur and lead to silent Duffy phenotype were not explored. Likewise, more than 200 mutations in the gene coding for G6PD have been described, some of which can result in different levels of enzyme deficiency [34]. Only the major *G6PD* genotypes associated with either African-type or Mediterranean-type deficiency were analyzed in our previous studies [33,40]. Second, PCR sequencing failed in a number of dried blood spots, most likely due to DNA degradation over time. The small quantity of available samples did not allow repetition of PCR amplification in case of PCR failure. However, the exclusion of some samples most probably did not introduce an important bias in sampling as all samples were subject to similar conservation conditions in the field. Third, data generated from the present study are not representative of the general population. Many of our blood samples were obtained from patients with symptoms suggestive of malaria. In the study site such as Atar, where white Moors predominate, patients infected with malaria or presenting with signs and symptoms associated with malaria are more likely to be white Moors, who tend to be overrepresented in the study area. In addition, no attempt was made to obtain a representative sample of each ethnic group because the Mauritanian government does not allow for a census identifying ethnic groups and the current ethnic composition of the country’s population is unknown. Fourth, data analysis by ethnic origin and study sites was not performed due to the tendency of some ethnic groups to assemble in certain regions of the country, with the exception of the capital city, the cosmopolitan melting pot of Mauritania. Fifth, at present, the original source of Duffy-negative and African-type *G6PD* A^–^ genotypes found in Mauritania today cannot be determined. Analysis of other gene markers, together with a more complete understanding of the history of human migration and interactions between human populations, would be necessary to gain a better knowledge of gene flow in Africa. Lastly, perhaps the most important limitation of the study from the clinical and epidemiological viewpoint is our inability to answer the question concerning the level of susceptibility of Duffy-negative white Moors and Duffy-positive individuals of black African ancestry to *P. vivax* malaria. Future studies designed to specifically answer these questions would be required.

## Conclusions

The novel multiplex amplicon-based DNA sequencing proved to be a rapid, high-throughput method to characterize Duffy genotype based on 11 major mutations in the present study, compared to the more labor-intensive PCR-RFLP or PCR sequencing using the Sanger dideoxy method to characterize *G6PD* genotype in our earlier works [33,40]. The novel methodological approach is useful for epidemiological studies. Further improvement of the multiplex assay for genotyping simultaneously Duffy and G6PD variants would enhance diagnostic efficacy. The results of the study showed two major populations: (1) mostly Duffy-positive white Moors with normal G6PD activity on one side of the spectrum and (2) mostly Duffy-negative ethnic groups of black African ancestry, some of whom presenting the African-type G6PD A^–^ deficiency, on the other side of the spectrum. The former is susceptible to *P. vivax* infection due to Duffy-positive phenotype, but most of them can be treated safely with primaquine; the latter is “resistant” to *P. vivax* malaria but, in case of infection, requires screening for G6PD activity before primaquine therapy. In between these two extremes are few individuals (Duffy-negative white Moors and Duffy-positive individuals of black African origin) who also require G6PD screening to ensure the safe administration of primaquine therapy. From the epidemiological viewpoint, the data presented here may be useful to identify priority areas in Mauritania to implement targeted interventions with primaquine, particularly in the southern Sahelian and Sahelian-Saharan transition zones.

## Supporting information

S1 Fig Sequencing depth and coverage of the amplicons. Mean sequence depth across individual samples is represented by the golden line (log 10 scale). The gray ribbon represents the 25^th^ and 75^th^ percentiles.
(TIF)

S1 Table Malaria prevalence in Mauritanian children and adults in five study sites located in different epidemiological strata. The prevalence rates are based on the number of PCR-positive blood samples (n) divided by the total number of included febrile patients (N) presenting spontaneously at one of our collaborating health centers between 2015 and 2020. Laboratory diagnosis was confirmed by using *Plasmodium* species-specific primers, as described in the cited references. Prevalence data in Atar, Nouakchott, Kobeni, and Rosso refer to PCR-confirmed diagnosis. ^1^ *Plasmodium falciparum* monoinfection. ^2^ *Plasmodium vivax* monoinfection and *P. falciparum*-*P. vivax* mixed infections. ^3^ The term “Black Africans” includes Black Moors and other ethnic groups of black African ancestry (Pulars, Soninkés, Wolofs). Two foreigners (1 Indian and 1 Malian) were inadvertently included in the study conducted in Atar, of whom 1 was PCR-positive (mixed infection). The total number of malaria-infected patients in Atar was modified to 453 – 2 = 451, with respect to the published data. ^4^ In Kobeni, a total of 2,040 and 286 Moors and Black Africans, respectively, were reported to have been included in the publication [55], of whom 45 had missing dried blood spots,. The total number of patients with PCR diagnosis was 2,281. In the published paper, the ethnic group “Moors” was defined by the linguistic criterion and included both White Moors and Black Moors, whereas the term “Black Africans” referred to ethnic groups of black African ancestry, with the exclusion of black Moors. To be consistent with the other publications referred to in this table, we separated “white Moors” from “black Moors” and considered black Moors to be part of the “Black African” ethnic groups. Few patients were shown to be infected by *P. malariae*, alone or in mixed infections. These cases were not included in this table.
(DOCX)

S2 Table Sequences of the adapter and primers used in the multiplex PCR targeted amplicon sequencing. Illumina Nextera universal tail sequences (in green) were added to the 5’-end of each primer to facilitate the library preparation in a two-step PCR approach. A barcode (in blue) and an additional adenine nucleotide (in orange) were inserted between the tail sequences and the primers to allow large-scale multiplexing of samples. bp, base pairs.
(DOCX)

S1 File Genotyping raw data for a set of 11 SNPs in ACKR1 gene associated with Duffy blood group system expression and its isoforms for 1,101 individuals from Mauritania. This file is derived from concatenated variant calling files (.vcf) for all individual. Each line corresponds to a genomic position at 11 selected loci on ACKR1 gene (RefSeq: NG_011626.3) for all individual. Sample: sample name. CHROM: reference genome on which sequence reads have been aligned on. POS: SNP position on the reference genome. REF: reference base at the position. ALT: alternate base at the position. QUAL: Phred-scaled quality score. GT: genotype with the allele values 0 for the reference allele (what is in the REF field) and 1 for the first allele listed in ALT so that 0_0 refers to samples homozygous reference, 0_1 refers to samples heterozygous (carrying one copy of each of the REF and ALT allele) and 1_1 refers to sample homozygous alternate. Phred-scaled genotype likelihoods rounded to the closest integer (PL) were given for all genotypes. The PL values of the most likely genotype (assigned in the GT field) is set to 0 in the Phred scale. Genotype: GT field modification to improve readability. Depth: read depth at this position. Run: identification of the sequencing run in which the sample was sequenced. All other information contained in the variant calling file are in the INFO field.
(XLSX)

S2 File R code used for the data visualization. The file provides code lines used to analyze the experimental data and perform data visualization and takes S1 File and S3 File as input files. This is an R Markdown file created using RStudio, an open-source Integrated Development Environment (IDE) for the R programming language. It contains YAML metadata, markdown-formatted plain text, and chunks of R code that can be rendered using RStudio.
(TXT)

S3 File Absolute frequencies of Duffy blood group system expression and its isoforms and G6PD expression in our cohort from Mauritania. Duffy blood group system expression and its isoforms were determined based on known polymorphic positions on ACKR1 gene and its promoter (Table 1). *G6PD* expression (g6pd +) and deficiency (g6pd -) were published in our earlier works [33,40] and based on the 2024 WHO classification [96]. X-linked *G6PD* deficiency cannot be genotypically determined in heterozygous females due to random X chromosome inactivation. Absolute frequencies were calculated as a function of the sex (F: female, M: male) and ethnic groups: Black Moors (BM), White Moors (WM), Pular (PL), Wolof (WF) and Soninke (SNK). NA refers to an absence of genotyping.
(XLSX)
