# Supplementary figures and images for: Amplicon-based DNA sequencing to characterize Duffy antigen polymorphisms and analysis of Duffy blood system and glucose-6-phosphate dehydrogenase deficiency in Mauritania
Source: PLoS Negl Trop Dis. 2025 Dec 26;19(12):e0013882. doi: 10.1371/journal.pntd.0013882 (PMC12768377; doi:10.1371/journal.pntd.0013882)

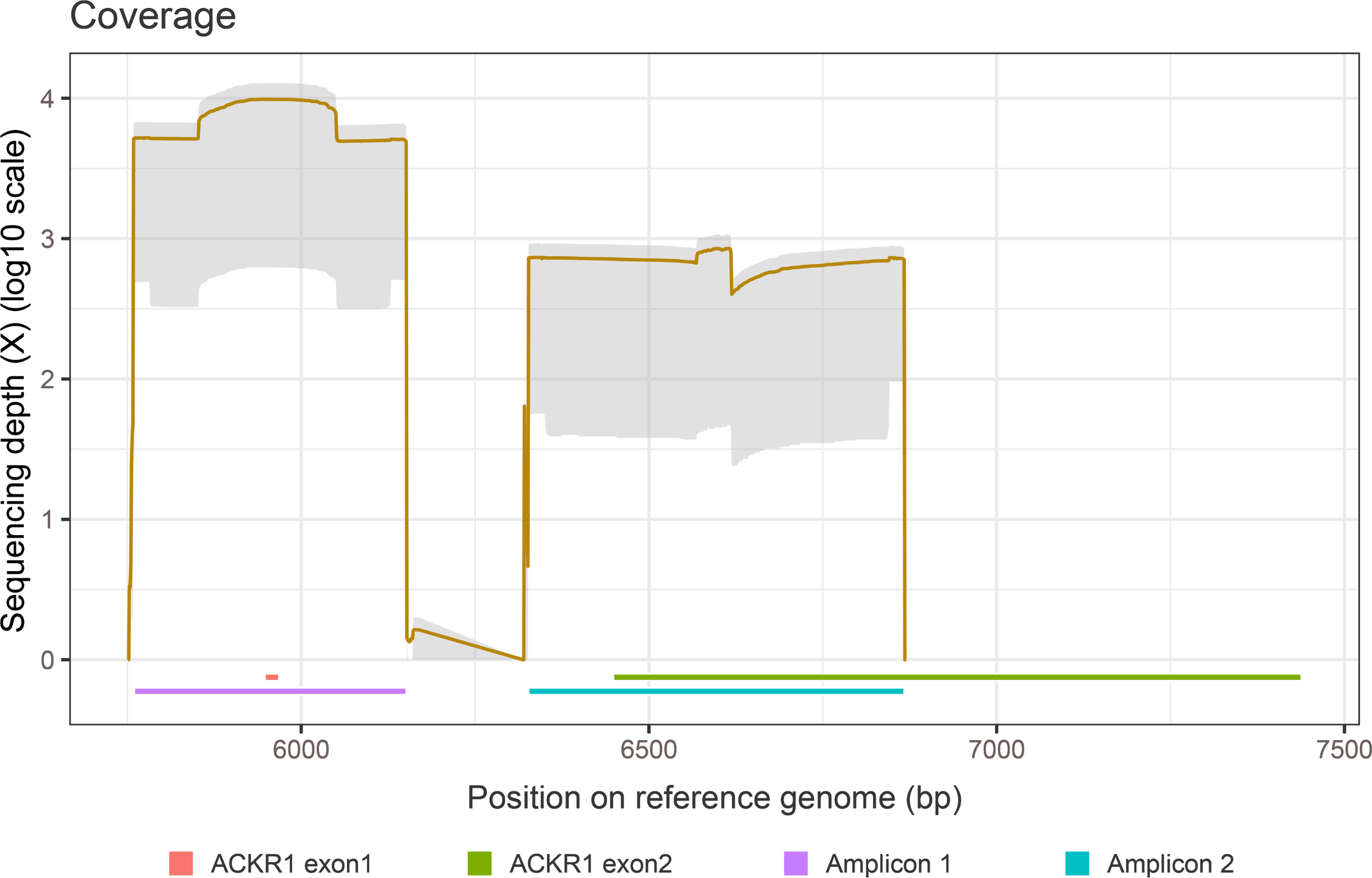

Supplement: S1 Fig — Mean sequence depth across individual samples is represented by the golden line (log 10 scale). The gray ribbon represents the 25th and 75th percentiles. (TIF) [file pntd.0013882.s001.tif]
