# Supplementary material for: Amplicon-based DNA sequencing to characterize Duffy antigen polymorphisms and analysis of Duffy blood system and glucose-6-phosphate dehydrogenase deficiency in Mauritania
Source: PLoS Negl Trop Dis. 2025 Dec 26;19(12):e0013882. doi: 10.1371/journal.pntd.0013882 (PMC12768377; doi:10.1371/journal.pntd.0013882)
Supplement: S1 Table — The prevalence rates are based on the number of PCR-positive blood samples (n) divided by the total number of included febrile patients (N) presenting spontaneously at one of our collaborating health centers between 2015 and 2020. Laboratory diagnosis was confirmed by using Plasmodium species-specific primers, as described in the cited references. Prevalence data in Atar, Nouakchott, Kobeni, and Rosso refer to PCR-confirmed diagnosis. 1 Plasmodium falciparum monoinfection. 2 Plasmodium vivax monoinfection and P. falciparum-P. vivax mixed infections. 3 The term “Black Africans” includes Black Moors and other ethnic groups of black African ancestry (Pulars, Soninkés, Wolofs). Two foreigners (1 Indian and 1 Malian) were inadvertently included in the study conducted in Atar, of whom 1 was PCR-positive (mixed infection). The total number of malaria-infected patients in Atar was modified to 453 – 2 = 451, with respect to the published data. 4 In Kobeni, a total of 2,040 and 286 Moors and Black Africans, respectively, were reported to have been included in the publication [55], of whom 45 had missing dried blood spots,. The total number of patients with PCR diagnosis was 2,281. In the published paper, the ethnic group “Moors” was defined by the linguistic criterion and included both White Moors and Black Moors, whereas the term “Black Africans” referred to ethnic groups of black African ancestry, with the exclusion of black Moors. To be consistent with the other publications referred to in this table, we separated “white Moors” from “black Moors” and considered black Moors to be part of the “Black African” ethnic groups. Few patients were shown to be infected by P. malariae, alone or in mixed infections. These cases were not included in this table. (DOCX) [file pntd.0013882.s002.docx]

**S1 Table. Malaria prevalence in Mauritanian children and adults in five study sites located in different epidemiological strata.**

| **Study site/ epidemiological strata** | **Ethnic group/study period** | **Prevalence (n/N, %)** | | **Reference** |
| --- | --- | --- | --- | --- |
|  |  | ***Plasmodium falciparum*^1^** | ***Plasmodium vivax*^2^** |  |
| Atar  Saharan zone oasis, northern Sahara | Overall (2015–2016)  White Moors  Black Africans^3^ | 4/451 (0.9)  4/352 (1.1)  0/ 99 (0) | 120/451 (35.0)  96/352 (27.2)  24/ 99 (24.2) | Deida et al., 2019 [38] |
| Nouakchott  Saharan zone, capital | Overall (2015–2020)  White Moors  Black Africans | 47/1760 (2.7)  33/1263 (2.6)  14/497 (2.8) | 216/1760 (13.9)  166/1263 (13.1)  50/497 (10.0) | El Moustapha et al., 2023 [39] |
| Kobeni^4^  southern Sahelian-Saharan transition zone | Overall (2015–2017)  White Moors  Black Africans | 1205/2281 (52.8)  353/ 722 (48.9)  852/1559 (54.6) | 147/2281 (6.4)  49/ 722 (5.5)  98/1559 (6.3) | Diallo et al., 2020 [55] |
| Rosso  Sahel, along the Senegal River basin | Overall (2015–2016)  White Moor  Black Africans | 0/318  0  0 | 2/318 (0.6)  1/227 (0.4)  1/ 91 (1.1) | Ould Lemrabott et al., 2021 [56] |

The prevalence rates are based on the number of PCR-positive blood samples (n) divided by the total number of included febrile patients (N) presenting spontaneously at one of our collaborating health centers between 2015 and 2020. Laboratory diagnosis was confirmed by using *Plasmodium* species-specific primers, as described in the cited references. Prevalence data in Atar, Nouakchott, Kobeni, and Rosso refer to PCR-confirmed diagnosis.

^1^ *Plasmodium falciparum* monoinfection.

^2^ *Plasmodium vivax* monoinfection and *P. falciparum*-*P. vivax* mixed infections.

^3^ The term “Black Africans” includes Black Moors and other ethnic groups of black African ancestry (Pulars, Soninkés, Wolofs). Two foreigners (1 Indian and 1 Malian) were inadvertently included in the study conducted in Atar, of whom 1 was PCR-positive (mixed infection). The total number of malaria-infected patients in Atar was modified to 453 – 2 = 451, with respect to the published data.

^4^ In Kobeni, a total of 2,040 and 286 Moors and Black Africans, respectively, were reported to have been included in the publication [55], of whom 45 had missing dried blood spots. The total number of patients with PCR diagnosis was 2,281. In the published paper, the ethnic group “Moors” was defined by the linguistic criterion and included both White Moors and Black Moors, whereas the term “Black Africans” referred to ethnic groups of black African ancestry, with the exclusion of black Moors. To be consistent with the other publications referred to in this table, we separated “white Moors” from “black Moors” and considered black Moors to be part of the “Black African” ethnic groups. Few patients were shown to be infected by *P. malariae*, alone or in mixed infections. These cases were not included in this table.
