# Supplementary material for: Amplicon-based DNA sequencing to characterize Duffy antigen polymorphisms and analysis of Duffy blood system and glucose-6-phosphate dehydrogenase deficiency in Mauritania
Source: PLoS Negl Trop Dis. 2025 Dec 26;19(12):e0013882. doi: 10.1371/journal.pntd.0013882 (PMC12768377; doi:10.1371/journal.pntd.0013882)
Supplement: S2 Table — Illumina Nextera universal tail sequences (in green) were added to the 5’-end of each primer to facilitate the library preparation in a two-step PCR approach. A barcode (in blue) and an additional adenine nucleotide (in orange) were inserted between the tail sequences and the primers to allow large-scale multiplexing of samples. bp, base pairs. (DOCX) [file pntd.0013882.s003.docx]

**S2 Table. Sequences of the adapter and primers used in the multiplex PCR targeted amplicon sequencing.**

| **Primer name** | **Sequence (5’ – 3’)** | **Length (bp)** |
| --- | --- | --- |
| Amplicon 1 (promoter region, 392 bp) | | |
| Duffy_promoter_tag1 | TCGTCGGCAGCGTCAGATGTGTATAAGAGACAGCATGAC**A**CCCAAGGCCAGTGACCCCCATA | 62 |
| Duffy_promoter_tag2 | TCGTCGGCAGCGTCAGATGTGTATAAGAGACAGTGCAGT**A**CCCAAGGCCAGTGACCCCCATA | 62 |
| Duffy_promoter_tag3 | TCGTCGGCAGCGTCAGATGTGTATAAGAGACAGACGTCA**A**CCCAAGGCCAGTGACCCCCATA | 62 |
| Duffy_promoter_tag4 | TCGTCGGCAGCGTCAGATGTGTATAAGAGACAGGTACTG**A**CCCAAGGCCAGTGACCCCCATA | 62 |
| Duffy_promoter_tag5 | TCGTCGGCAGCGTCAGATGTGTATAAGAGACAGCTAGTC**A**CCCAAGGCCAGTGACCCCCATA | 62 |
| Duffy_promoter_tag6 | TCGTCGGCAGCGTCAGATGTGTATAAGAGACAGAGCTGA**A**CCCAAGGCCAGTGACCCCCATA | 62 |
| Duffy_promoter_tag7 | TCGTCGGCAGCGTCAGATGTGTATAAGAGACAGTCGACT**A**CCCAAGGCCAGTGACCCCCATA | 62 |
| Duffy_promoter_tag8 | TCGTCGGCAGCGTCAGATGTGTATAAGAGACAGGATCAG**A**CCCAAGGCCAGTGACCCCCATA | 62 |
| Duffy_promoter_tag9 | TCGTCGGCAGCGTCAGATGTGTATAAGAGACAGATGCTA**A**CCCAAGGCCAGTGACCCCCATA | 62 |
| Duffy_promoter_tag10 | TCGTCGGCAGCGTCAGATGTGTATAAGAGACAGGCATAG**A**CCCAAGGCCAGTGACCCCCATA | 62 |
| Duffy_promoter_tag11 | TCGTCGGCAGCGTCAGATGTGTATAAGAGACAGCGTAGC**A**CCCAAGGCCAGTGACCCCCATA | 62 |
| Duffy_promoter_tag12 | TCGTCGGCAGCGTCAGATGTGTATAAGAGACAGTACGCT**A**CCCAAGGCCAGTGACCCCCATA | 62 |
| Duffy_promoter_reverse | GTCTCGTGGGCTCGGAGATGTGTATAAGAGACAGAGAGGGAGCTAGGAGGCTAGCAT | 57 |
| Amplicon 2 (exon 2) | | |
| Duffy_tag1 | TCGTCGGCAGCGTCAGATGTGTATAAGAGACAGCATGAC**A**CCTGCAGAGACCTTGTTCTCCCAC | 64 |
| Duffy_tag2 | TCGTCGGCAGCGTCAGATGTGTATAAGAGACAGTGCAGT**A**CCTGCAGAGACCTTGTTCTCCCAC | 64 |
| Duffy_tag3 | TCGTCGGCAGCGTCAGATGTGTATAAGAGACAGACGTCA**A**CCTGCAGAGACCTTGTTCTCCCAC | 64 |
| Duffy_tag4 | TCGTCGGCAGCGTCAGATGTGTATAAGAGACAGGTACTG**A**CCTGCAGAGACCTTGTTCTCCCAC | 64 |
| Duffy_tag5 | TCGTCGGCAGCGTCAGATGTGTATAAGAGACAGCTAGTC**A**CCTGCAGAGACCTTGTTCTCCCAC | 64 |
| Duffy_tag6 | TCGTCGGCAGCGTCAGATGTGTATAAGAGACAGAGCTGA**A**CCTGCAGAGACCTTGTTCTCCCAC | 64 |
| Duffy_tag7 | TCGTCGGCAGCGTCAGATGTGTATAAGAGACAGTCGACT**A**CCTGCAGAGACCTTGTTCTCCCAC | 64 |
| Duffy_tag8 | TCGTCGGCAGCGTCAGATGTGTATAAGAGACAGGATCAG**A**CCTGCAGAGACCTTGTTCTCCCAC | 64 |
| Duffy_tag9 | TCGTCGGCAGCGTCAGATGTGTATAAGAGACAGATGCTA**A**CCTGCAGAGACCTTGTTCTCCCAC | 64 |
| Duffy_tag10 | TCGTCGGCAGCGTCAGATGTGTATAAGAGACAGGCATAG**A**CCTGCAGAGACCTTGTTCTCCCAC | 64 |
| Duffy_tag11 | TCGTCGGCAGCGTCAGATGTGTATAAGAGACAGCGTAGC**A**CCTGCAGAGACCTTGTTCTCCCAC | 64 |
| Duffy_tag12 | TCGTCGGCAGCGTCAGATGTGTATAAGAGACAGTACGCT**A**CCTGCAGAGACCTTGTTCTCCCAC | 64 |
| Duffy_reverse | GTCTCGTGGGCTCGGAGATGTGTATAAGAGACAGAGCAGCAAAGCCTGGGCAAAGG | 56 |

Illumina Nextera universal tail sequences (in green) were added to the 5’-end of each primer to facilitate the library preparation in a two-step PCR approach. A barcode (in blue) and an additional adenine nucleotide (in orange) were inserted between the tail sequences and the primers to allow large-scale multiplexing of samples. bp, base pairs.
